# CRYPTIC TRANSCRIPTION IS ASSOCIATED WITH AGE IN MAMMALIAN STEM CELLS

**DOI:** 10.1093/geroni/igz038.3493

**Published:** 2019-11-08

**Authors:** Brenna McCauley, Luyang Sun, Weiwei Dang

**Affiliations:** Baylor College of Medicine, Houston, Texas, United States

## Abstract

Aging is a multifaceted process that challenges organisms with stresses resulting from the dysregulation of cellular processes. Unsurprisingly, given how tightly regulated it is under normal conditions, transcription is one of the key pathways disrupted during aging. Indeed, dysregulation of transcription contributes to the activation of transposable elements, the loss of cellular identity, and decreased stem cell potency with age. Our previous work identified intragenic cryptic transcription (CT) as a novel type of age-associated transcriptional dysregulation that limits the lifespan of yeast and worms. Continuing this work, we show for the first time that CT increases with age in mammalian stem cells. Increased CT is associated with disrupted chromatin structure, particularly with the reduction of H3K36me3, a histone modification known to inhibit CT throughout eukaryotes. We propose that an age-associated reduction in H3K36me3 in actively transcribed gene bodies drives disruption of chromatin structure in these regions, resulting in an open chromatin state. This open chromatin state is permissive for the entry of RNA Pol II, which can then initiate transcription from within the gene body. These aberrant cryptic transcripts may contribute to the pathological load of mammalian aging.

